# Current News and Opinion

**Published:** 1887-03

**Authors:** 


### Current News and Opinion.

NINTH INTERNATIONAL MEDICAL CONGRESS, WASHINGTON, D. C., SEPTEMBER 5, 1887.

*President*—N. S. Davis, M. D., LL. D.

*Secretary General*—John B. Hamilton, M. D. of U. S. A.

SECTION 17—DENTAL AND ORAL SURGERY.

*President*—Dr. J. Taft.

*Vice-Presidents.*

Dr. W. W. Allport,
Dr. F. Abbott,
Dr. W. C. Barrett,
Dr S. W. Dennis,
Dr. C. L. Ford,
Dr. W. H. Morgan,
Dr. H. J. McKellops,
Dr. A. T. Metcalf,
Dr. A. L Northrop,
Dr. A. O. Rawls,
Dr. Joseph Richardson,
Dr. C. W. Spalding,
Dr. L. D. Shepard,
Dr. James Truman,
Dr. W. W. H. Thackston,
Dr. V. E. Turner,
Prof. Dr. F. Busch,
Dr. W. Herbst,
Dr. L. N. Hollander,
Dr. Andrieu,
Dr. E. Magitot,
Dr. V. Haderup,
Dr. T. H. Harding,
Dr. W. Geo. Beers.

*Secretaries.*

Dr. E. A. Bogue, 29 East 20th Street, ........................ New York City.
Dr. F. H. Rehwinkel ........................................ Chillicothe, Ohio.
Dr. E. Brasseur, 6 Rue Mogador ................................ Paris, France.
Dr. Elof Fœrberg ........................................ Stockholm.
Dr. Julius Parreidt ........................................ Leipzig, Germany.

*Council.*

Dr. R. R. Andrews,
Dr. C. F. W. Bœdecker,
Dr. C. A. Brackett,
Dr. B. H. Catching,
Dr. George H. Chance,
Dr. E. S. Chisholm,
Dr. C. C. Chittenden,
Dr. D. M. Clapp,
Dr W. R. Clifton,
Dr. J. S. Cassidy,
Dr. K. B. Davis,
Dr. A. M. Dudley,
Dr. M. W. Foster,
Dr. J. G. Friedrichs,
Dr. C. E. Francis,
Dr. George L. Field,
Dr. F. J. S. Gorgas,
Dr. P. G. C. Hunt,
Dr. A. O. Hunt,
Dr. R. Finley Hunt,
Dr. George W. Keely,
Dr. Edmund C. Kirk,
Dr. James Lewis,
Dr. James McManus,
Dr. W. N. Morrison,
Dr. J. Hall Moore,
Dr. T. T. Moore,
Dr. Edgar Palmer,
Dr. H. J. Plomteaux,
Dr. S. B. Palmer,
Dr. W. A. Spalding,
Dr. C. S. Stockton,
Dr. A. H. Thompson,
Dr. W. C. Wardlaw,
Dr. J. W. White.

*Finance Committee of this Section.*

| | |
|---|---|
| Dr. J. W. White, Treasurer, | Dr L. D. Shepard, |
| Dr. A. M. Dudley, Secretary, | Dr. C. H. Winkler, |
| Dr. Edgar Palmer, | Dr. W. W. Allport, |
| Dr. H. J. McKellops, | Dr. W. W. Walker. |

*Reception Committee of this Section.*

| | |
|---|---|
| Dr. A. L. Northrop, | Dr. D. McFarlan, |
| Dr. H. J. McKellops, | Dr. James McManus, |
| Dr. A. M. Dudley, | Dr. W. W. H. Thackston, |
| Dr. William Carr, | Dr. E. A. Bogue, |
| Dr. L. D. Shepard, | Dr. F. H. Rehwinkel, |
| Dr. E Maynard, | Dr C. F. W. Bœdecker. |

*Committee on Operative Dentistry and Oral Surgery—Clinics.*

| | |
|---|---|
| Dr. C. F. W. Bœdecker, | Dr. F. Abbott, |
| Dr. J. A. Watling, | Dr. W. C Wardlaw, |
| Dr. R. L. Cochran, | Dr J. D. Patterson. |

*Committee on Prosthetic Dentistry.*

| | |
|---|---|
| Dr. George L. Field, | Dr. John Allen, |
| Dr. H. B. Noble, | Dr. T. T. Moore, |
| Dr. A. O. Hunt, | Dr. N. W. Morrison, |

Dr. R. B. Donaldson.

---

### CASES OF SKIN ERUPTIONS AND SYPHILIS TREATED WITH HORSFORD'S ACID PHOSPHATE.

It appears to me that the "Acid Phosphate" originally prescribed by Prof. Horsford, of Cambridge, U. S. A., is not so well known in this country as its merits deserve. A glance at the formula will, however, readily convince one of its value in suitable cases. Each fluid drachm gives on analysis five and one-half grains of free phosphoric acid, and nearly four grains of phosphate of lime, magnesia, iron and potash. The following are a few brief notes of some of the cases in which I have prescribed it with complete success.

Mr. G., æt. 69, consulted me November, 1885, for eczema on the arms, legs, palms of the hands, and trunk. The patient complained of much debility and nervous exhaustion, and he was a man who had led a very busy business life, with much worry. In December, 1885, I prescribed Horsford's acid tonic with much good effect, as in February, 1886, I heard that he was quite well.

Mrs. S., æt. 46, consulted me in December, 1885, for psoriasis, all over the body, more or less, especially on the legs and arms. In January, 1886, I prescribed a teaspoonful of the acid tonic three times a day with marked good effect. Patient had been much exhausted by continuous nursing of an invalid mother.

Mr. C., æt. 64, consulted me in September, 1885, with one of the worst attacks of late syphilis I ever saw. After he had been relieved from the distressing symptoms and ulcerations, I prescribed the acid tonic for epileptiform fits from which he suffered, with excellent results.

Mr. McJ., æt. 63, consulted me in November, 1885, for lichen ruber, which was accompanied with intolerable itching. He was a nervous, irritable man. I prescribed the acid tonic with the effect that in December he presented himself quite convalescent.—*James Startin, Medical Fress, London, Eng.*

### DENTAL JOURNALS WANTED.

The editor of this journal will pay cash for the following numbers of dental journals, or an exchange will be made with those who desire to complete their own files:

THE DENTAL REGISTER.

Vol. III , Nos. 1, 2, 3.

Vol. VI., Nos. 1, 2, 3, 4.

AMERICAN JOURNAL OF DENTAL SCIENCE, (THIRD SERIES.)

Vol. II., No. 8.
Vol. VII., Nos 7, 10.
Vol. VIII., Nos 6, 7.

### CHICAGO DENTAL CLUB

The Chicago Dental Club held its annual meeting at the Tremont House, Monday evening, January 24th, when the following officers were elected :

*President*—L. P. Haskell, D. D. S.
*Vice-President*—John S. Marshall, M. D.
*Secretary*—Arthur B. Freeman, M. D., D. D. S.
*Treasurer*—Dr. E. M. S. Fernandez.
*Business Committee*—Eugene S. Talbot, M. D., D. D. S ; Dr. Austin Freeman and Charles P. Pruyn, M. D., D. D. S.

Very courteously yours,
ARTHUR B. FREEMAN,
Secretary C. D. C.

### VERMONT STATE DENTAL SOCIETY.

The eleventh annual meeting of the Vermont State Dental Society will be held at the Van Ness House, Burlington, Vt., Wednesday, March 16, 1887, commencing at 7.30 P. M. and continuing through Friday.

Members of the profession in this State and others are cordially invited to be present, and are requested to bring specimens or models of cases in practice, and time for this exhibition will be given. The depots will exhibit their supplies as usual. Railroads will give return checks.

THOS MOUND, Secretary,

THE NEW YORK MEDICAL JOURNAL says that the struggles of young doctors are hard enough, as few, if any, of our readers need to be reminded. To those who are inclined to regard their own prospects as gloomier than if they had chosen some other profession, we commend an advertisement that lately appeared in *The Daily Register*, the New York law journal, in which "an experienced attorney" offered his services to any law firm at a salary of five dollars per week.

The Purity of Mid-Atlantic Air.—In the course of an address on the action of micro-organisms on surgical wounds, Prof. F. S. Dennis, of New York, states that during his last trip across the Atlantic he made some experiments to test the purity of the air 1,000 miles from land. He employed capsules of sterilized gelatine, and exposed them for fifteen minutes. One capsule was exposed in the state-room upon the main deck of the steamer. Within eighteen hours 500 points of infection had developed. Two capsules exposed in a similar manner in a cabin on the promenade deck, where the circulation of the air was free, showed five or six points of infection ten days afterward. A capsule exposed over the bow of the ship was found to be entirely uncontaminated. These experiments are on the same line with those of Pasteur and Tyndall upon the mountain air of Switzerland; and, so far as they go, they show the germless condition of mid-ocean air, and also the need for much more efficient ventilation in the state-rooms of even the first-class American lines.—*Lancet.*

---

Certain Alienists, notably Moreau de Tours, have claimed that genius, especially poetic genius, is a neurosis, and that the man of genius is but a

Poor lunatic, who makes his moan,
And for a while beguiles his lookers-on.
He reasons well; his eyes their wildness lose,
But if you hit the cause that hurts his brain
His eyeballs roll, and he is mad again.

Nathaniel Lee, the periodical lunatic, who wrote these lines, had very good reason for accepting this theory. Nor is Nathaniel Lee the only mad poet. Lucretius, Marlowe, Ben Jonson, Bunyan, Wycherly, Tarquato Tasso, Molière, Swift, Pope, De Foe, Rousseau, Goldsmith, Johnson, Savage, Cowper, Byron, Walter Scott, Coleridge, De Quincey, Rogers, Southey, Shelley, Emerson, Saxe, Cobb and Victor Hugo, all suffered from insanity.

Poetry is an emotional outburst of a perception of similarities in unlike things. Hence the "fine poetic frenzy" of the poet is not unlike the emotional exaltation of the insane.—*Neurological Review.*

---

At the Annual Meeting of the Central Dental Association of Northern New Jersey, held at 764 Broad Street, Newark, N. J., February 21, 1887, the following officers were elected:

*President*—S. C. G. Watkins, Montclair.
*Vice-President*—George E. Adams, South Orange.
*Secretary*—James G. Palmer, New Brunswick.
*Treasurer*—Charles A. Meeker, Newark.

*Executive Committee.*

B. F. Luckey, Paterson, Oscar Adelberg, Elizabeth,
C. F. Holbrook, Newark, Wm. P. Richards, Orange,
Jacob Simonson, Newark.

James G. Palmer, Secretary.

It will always be hard to understand how the thousands of tons of snow which fall in a single storm can be suspended in the upper regions of the atmosphere. We know the snow is formed from the condensation and freezing of aqueous vapor, or steam; but when we learn that a cubic foot of snow which falls is condensed from 1,728 cubic feet of aqueous vapor, the amount necessary to form the snow of a single storm is almost beyond one's powers of calculation.

With condensation of snow from aqueous vapor, an immense amount of so-called latent heat is set free. In the condensation of a single pound of steam to water, enough heat is evolved to raise a pound of water from 0° to 972° Fahrenheit, and an additional amount cf heat is given out when the water is frozen into snow or ice. It may be said that a fall of snow is equivalent to a fall of red hot sand as far as the heat produced in its formation is concerned; and although this snow may not produce any appreciable effect upon the temperature of the air at the surface of the earth, yet it shows the tremendous energy of the operations taking place in the upper air, and the enormous amount of cooling necessary to produce a single fall of rain or snow.—*Popular Science News.*

---

The *Revue Scientifique* claims the first thought of the germ theory of disease for a Dr. Goiffon, who died at Lyons more than 150 years ago. He believed, in 1721, that diseases, like a plague, could be caused only by minute insects or worms, too small to be seen perhaps, but nevertheless really existing. He also believed that the conveyance of infection could be explained by their activity and propagation.—*Medical News.*

It should be comprehended that the germ theory of diseaes does *not* claim the existence of "minute insects or worms" as the cause of disease. It is only ignorant people who suppose this. Micro-organisms are of a vegetable nature, rather than animal. They are microscopical *fungi.*—Editor.

---

Prof. Sergei Petrovich Kolomin, Professor of Surgery in the University of St. Petersburg, and one of the most brilliant surgeons in Russia, committed suicide on the 23d of November last, by shooting himself in the head with a pistol. In operating on a female patient for what he took to be a tubercular disease of the rectum, he injected into the viscus twenty-four grains of cocaine, which caused the patient's death. The autopsy showed that the diagnosis was a mistaken one. The affair so preyed upon the mind of Prof. Kolomin, that he resorted to suicide for relief.

---

A new journal has just appeared in Germany, entitled the *Centralblatt fuer Bacteriologie and Parasitenkunde.* It is published weekly, and will be devoted to all that pertains to the study of bacteriology. The editors are Dr. Oscar Uhlworm, of Cassel, Dr. Lenchart, of Leipzig, and Dr. Lœffler, of Berlin. The publisher is Gustav Fisher, of Jena. From the two issues that have thus far appeared, we should judge that it will be a welcome guest in America as well as in Germany, especially to those who can read the German.

IT IS A CAUSE OF REGRET to know that Dr. J. S. Jewell, who projected and published the first numbers of a new journal of psychiatry called *The Neurological Review*, has, on account of ill-health, been compelled to suspend it for the present. The known ability of Dr. Jewell and his thorough knowledge of his specialty gave assurance of an excellent journal, and the few numbers issued were a demonstration and foretaste of the excellence that was to have characterized the new journal.

SAID A LADY PATIENT the other day, "I always know before I get up in the morning whether the day is to be a pleasant or stormy one "

"Indeed," was our reply, "pray state the indications ?"

"Well, the gentleman residing in next house is a pretty good liver, and has the gout. If a storm is approaching, he groans dreadfully all night, but if the weather is to be clear he is quiet." F.

DR. L. C INGERSOLL'S "Questions and Answers in Dental Science" is meeting with an unusual measure of success, and has gained the unqualified approval of many of the best men in dentistry. For a book that was so modestly issued, it has attracted a great deal of attention. Dr. Ingersoll is himself a thinking man, and to any one who reasons for himself his writings have an irresistible charm.

THE JANITOR of a very dirty suite of offices had posted up at the entrance the notice: "Visitors will please wipe their feet."

A practical joker, after having inspected the apartments one day, added the following : "On going out."

WARTS MAY BE CURED, says a German medical journal, by brushing them carefully once a day with a solution of fifteen grains of corrosive sublimate in one ounce of collodion. This remedy is said to be more efficacious, and it is certainly more convenient, than many other preparations recommended.

DR. A M. DUDLEY has been presented by Provincetown Post, G. A. R., with a cane of historic interest, as a mark of their appreciation of his interest in their organization.

DR. GEORGE L FIELD, of Detroit, Mich , is about these times taking a vacation in Florida, in the land of the myrtle and the palm, of the mosquito and the alligator.

"THE DOCTOR" is the name of a new semi-monthly medical journal published in New York. It is very sprightly and as full of original matter as a new-laid egg.

CREOSOTE three parts and collodion two parts, when mixed, make a solid mass which is very convenient for office use.

STEEL, when hardened, decreases in specific gravity, contracts in length, and increases in diameter

LAVOISIER, the famous French chemist, was guillotined by his countrymen.

